# Mantle-derived fluid flux controls Olympic Dam-style Fe oxide-Cu-Au mineralisation

**DOI:** 10.1038/s41598-025-33477-7

**Published:** 2026-01-16

**Authors:** Stephan Thiel, Anthony Reid, Graham Heinson, Kate Brand, Lu Li

**Affiliations:** 1 CSIRO, Mineral Resources, Urrbrae, 5064 SA Australia; 2 Fleet Space Technologies, 28 Butler Boulevard, Adelaide Airport, 5950 Adelaide, Australia; 3 School of Physics, Chemistry and Earth Sciences, The University of Adelaide, 5005 Adelaide, SA Australia; 4 Bureau of Meteorology, Adelaide, Australia; 5 CSIRO, Mineral Resources, CSIRO, Kensington, WA 6151 Australia

**Keywords:** Magnetotellurics, Mineral exploration, Craton lithosphere, Metasomatism, Gawler craton, Geomagnetism, Geophysics, Solid Earth sciences, Tectonics

## Abstract

Conventional mineral exploration has focused on processes in the mid-upper crust, however recent advances in the geophysical and geochemical understanding of the sub-continental lithospheric mantle (SCLM) suggest that the macroscopic architecture of the lithosphere plays a key role in the localization of giant mineral deposits. Here, we use AusLAMP (Australian Lithospheric Architecture Magnetotelluric Project) magnetotelluric (MT) data to image the footprint of an entire mineral system, the Mesoproterozoic iron oxide copper-gold (IOCG) province of the eastern Gawler Craton, Southern Australia. Our new 3D resistivity model demonstrates a physical connection exists between the anomalous, enriched SCLM beneath the Gawler Craton and the detailed resistivity mapping of the upper and middle crust beneath the Olympic Dam IOCG deposit. This conductivity network represents a whole of lithosphere plumbing system, which constitutes a direct pathway from a mantle source at the margins of an Archean cratonic core to form the metallogenic province at the surface. We argue that primary controls of lithospheric architecture and optimal crustal conditions, including high strain localization and secondary fluid availability, are required for large scale thermal events to provide the necessary low-entropy physico-chemical environment for deposition of large IOCG type deposits in the crust.

## Mapping of large-scale mineral systems

Mineral exploration is typically reliant on ore deposit models from known deposits coupled with surface mapping, geochemistry, potential field and shallow sensing electrical geophysics for direct targeting in the upper crust. Nevertheless, there is a growing recognition that in poorly explored and especially covered greenfield terranes, the first stage of the exploration scale-reduction process must necessarily focus upon detection of the mineral system as a whole^2^, rather than detecting mineral deposits per se. One of the crucial ingredients in the formation of many large metallogenic provinces is a high energy flux driven by the ascent of magmas into and within structural corridors in the crust that often originate with thermo-mechanical perturbation in an anomalously hydrous lithospheric mantle^3–5^. Geophysical methods that can image the entire lithosphere are therefore required in order to detect this aspect of the mineral system.

Recent studies suggest the importance of lithosphere-scale processes involving a re-fertilized SCLM^6–8^ for the formation of base and precious metal provinces. Strong correlation between the location of copper deposits and the margins of cratonic lithospheric blocks^6,9,10^, the occurrences of native Au within mantle rocks^7^, and the importance of hydrated mantle pyroxenites for Ni-sulfide systems^11,12^ highlight the importance of recognizing re-fertilized SCLM in the genesis of large metallogenic provinces. To date, studies have focused primarily on mapping the bulk physical properties of the SCLM using seismic tomography^13–15^ and isotope geochemistry^7^ showing the genetic links between metals and the underlying re-fertilized SCLM^8^. There are, however, no conclusive examples of whole-of-lithosphere geophysical imaging of the crucial link of the geometry of the magmatic and fluid pathways that drive the entire mineral system^16^, from the SCLM to a deposit in a world-class mineral province^17^.

There are two main geophysical methods capable of imaging the Earth at an entire-lithosphere scale, passive seismic and magnetotelluric methods. While seismic tomography studies map bulk elastic properties of the lithosphere, the metasomatic footprint of ancient magmatic events and fluid flux that have cooled to solid state is largely represented only by minor mineralogical phases along grain boundaries and hydration of nominally anhydrous minerals^18^. In the absence of present-day partial melts, seismic shear-wave receiver functions and marginal seismic wave speed perturbations have been interpreted to result from the presence of zones of hydration along mid-lithosphere discontinuities, that is, by metasomatism^8,19^.

Minor conducting phases due to the hydration of the SCLM have a significant effect on bulk electrical resistivity^20^, at times by several orders of magnitude^21^. Laboratory measurements show a decrease in electrical resistivity as a result of hydrogen in the crystal lattice of mantle-constituting minerals, such as olivine^22,23^, pyroxenes^24,25^ and garnet^26^. For this reason, the MT method, measuring the variation of the naturally occurring electric and magnetic field of the earth to map the bulk electrical resistivity distribution of the SCLM and overlying crust, is an ideal tool to map metasomatic mineral system footprints in a given piece of lithosphere.

Here, we present a new craton-scale 3D resistivity model of 282 long-period (modelled across 24 periods in a range represented by most stations from 10 s - 10.000 s) MT stations across an area of $1200\times 700 \, \textrm{km}$ in southern Australia. This data is part of the AusLAMP project, which aims to map the electrical structure of the entire Australian continent. The new 3D model reveals that the IOCG-type deposits in the Gawler Craton have an electrical mineral system footprint spanning the entire lithospheric column, requiring whole-of-lithosphere geodynamic processes to explain their genesis. We demonstrate the importance of a re-fertilized SCLM, as well as the influence of cratonic margins for the location of IOCG deposits.

## Structure and evolution of the Gawler Craton

**Fig. 1:**
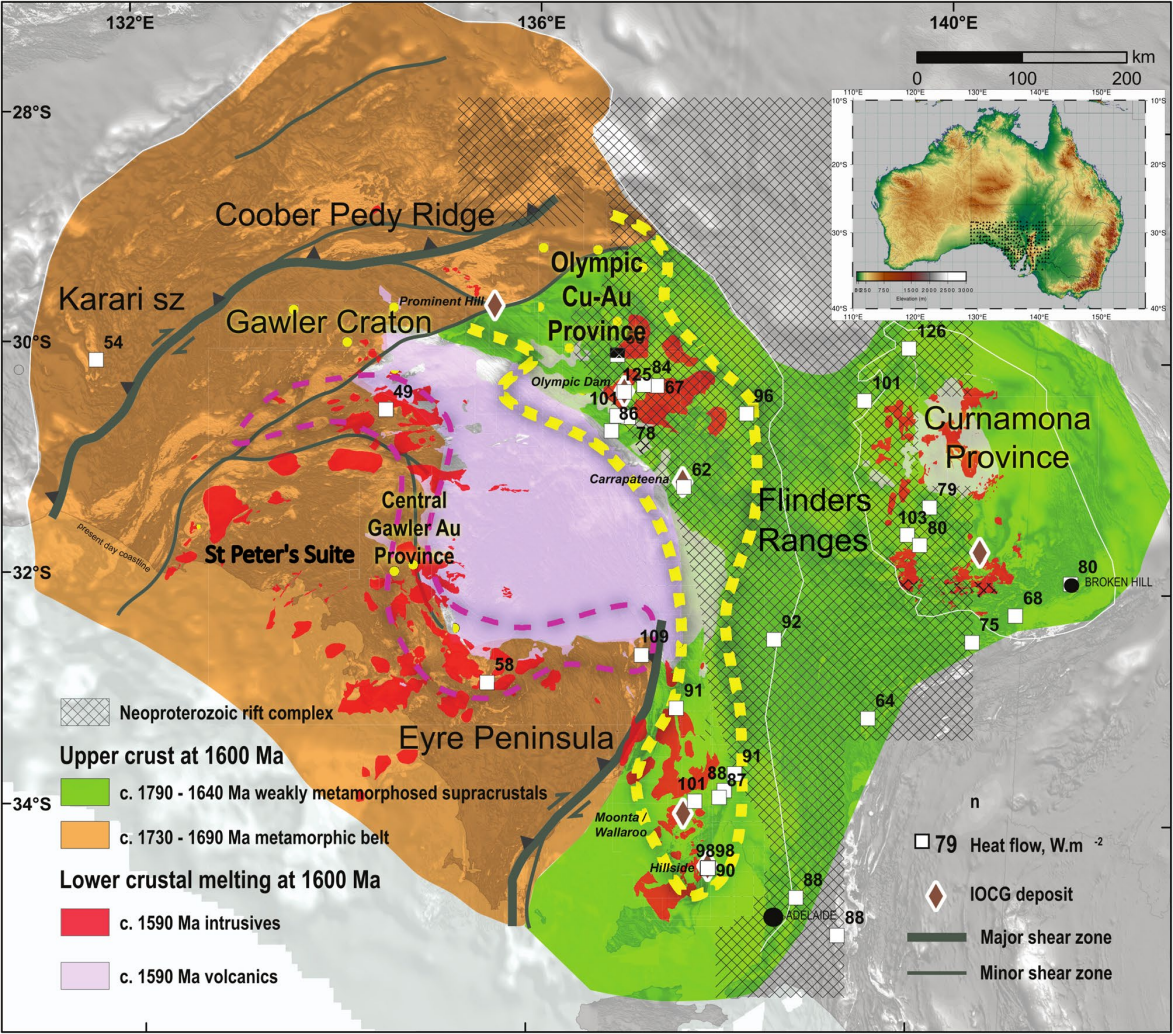
The iron-oxide copper gold (IOCG) province of the Olympic Province in the context of the geology of the Gawler Craton and Curnamona Province. The ca. 1590 Ma intrusives and volcanics shown in context of the distribution of higher metamorphic grades found in the central-western Gawler Craton in comparison to lower metamorphic grades in the eastern Gawler Craton^1^. Yellow dashed line denotes the Olympic Cu-Au Province. Heat flow values are shown as white squares. Inset shows the location of the array on a digital elevation model of Australia.

Although typically viewed as a coherent block of Archean to Proterozoic stable continental lithosphere, the Gawler Craton preserves a remarkable heterogeneity in several independent geological and geophysical datasets. These datasets suggest that the lithosphere can be viewed as a composite of two major lithospheric blocks, with the junction between these being located within the eastern region of the craton broadly corresponding to the eastern edge of the Olympic Cu-Au Province shown in Fig. 1.

Seismic tomography shows the lithosphere-asthenosphere boundary (LAB) to be deepest in the region of the central Gawler Craton, with depths up to 220 km indicated in the AusREM model^14,27^. Modern day heat flow data from across the Gawler Craton shows a 1.5 to 2 times greater heat flux in the region of the eastern Gawler Craton than the equivalent in the central regions of the craton^28^ (Fig. 1). The elevated heat flow of the eastern Gawler Craton is indicative of the greater upper crustal heat production in this region and is suggestive of a difference in bulk geochemical composition. The crustal heat production budget can be modified and concentrated via magmatism and orogenesis; themselves a function of bulk lithospheric scale rheology and melt fertility^28,29^.

Whole rock Sm-Nd isotopic data for felsic igneous rocks across the Gawler Craton shows a pronounced gradient from the central Gawler Craton towards the east^30^. The central Gawler Craton and regions of the Eyre Peninsula preserve Archean rocks with two-stage depleted mantle model ages up to c. 2.9 Ga^31^. In contrast, in the region around Olympic Dam and the broader eastern Gawler Craton, model ages are predominantly younger, c. 2.1 to 1.9 Ga. This range in model age may reflect that the western and central Gawler Craton are underlain by Archean lithosphere^32^. The Sm-Nd isotopic data also highlight the central Gawler Craton as containing a region of juvenile crust; corresponding largely to the c. 1.62 Ga magmatic St. Peter Suite^30^.

Major mineralizing events are preserved in the Gawler Craton at c. 1.59 Ga represented by the Olympic Cu-Au Province and a series of Au-dominated, yet polymetallic deposits in the central Gawler Craton that have been linked via the concept of the Central Gawler Gold Province^33^.

The orogenic history of the Gawler Craton prior to the c. 1.59 Ga event also reflects contrasts in rheological behavior, most strikingly manifest as the variation in behavior during orogenesis. While at the current exposure level, the eastern Gawler Craton appears to have escaped significant deformation at c. 1.7 Ga, the remainder of the craton experienced high-temperature moderate pressure metamorphism and associated deformation. This has been argued to have influenced the types of fluids available for ore-forming processes during the subsequent c. 1.59 Ga event^1^. Subduction-related metasomatism of the mantle lithosphere has been suggested either through subduction prior to the 1.59 Ga event^8,34^ or through a supra-subduction scenario^35^.

Therefore, the abundance of metamorphic fluids and the location near the edge of the craton renders eastern Gawler craton a favourable environment for mineral deposits, especially in light of a metasomatised mantle as further outlined below.

**Fig. 2:**
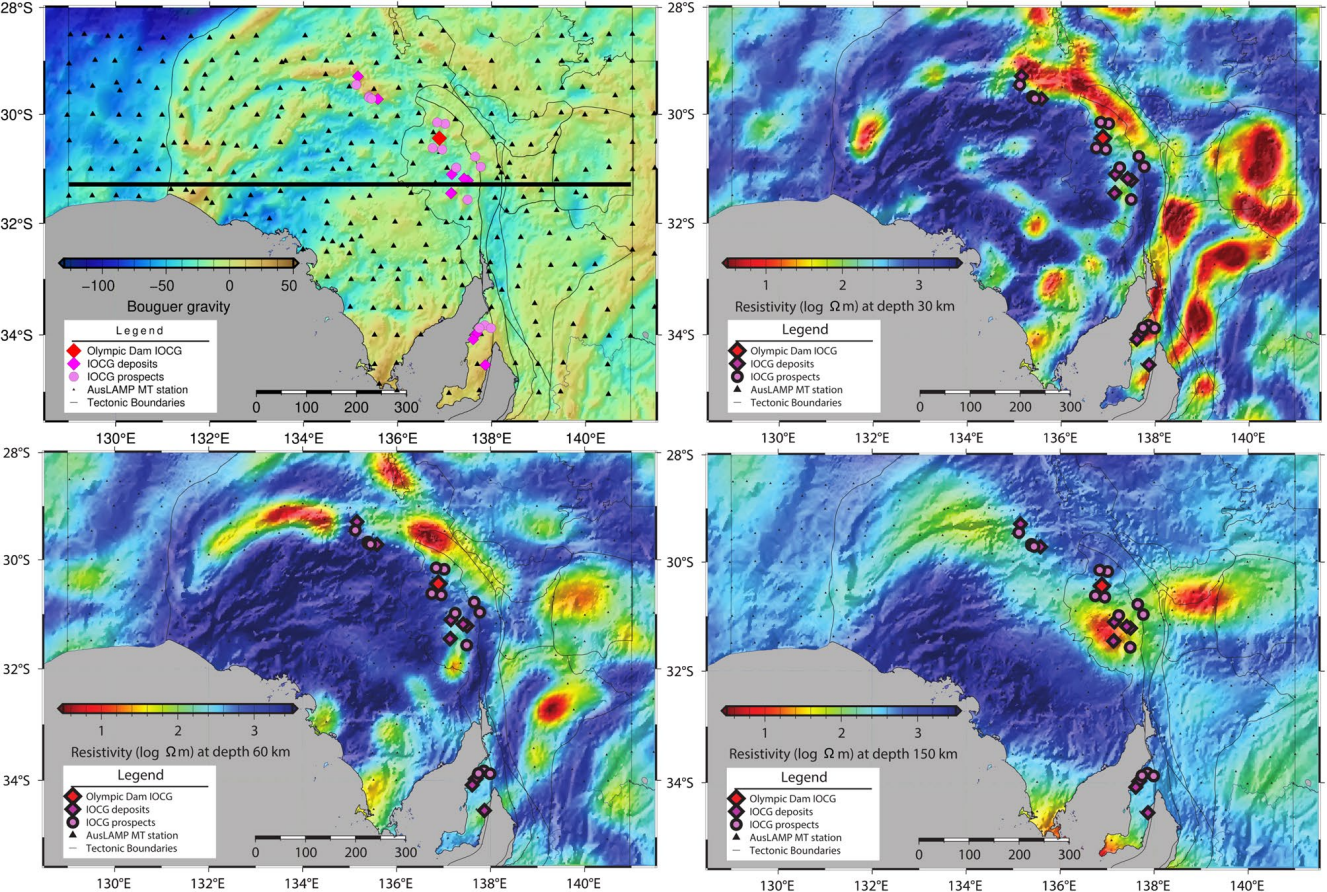
Geophysical responses of the study area superimposed with tectonic outlines and IOCG deposits and prospects. From top left to bottom right: Bouguer gravity with deposits superimposed and east-west profile of the cross-section in Fig. 3, MT resistivity depth slice with surface Bouguer gravity response shading at 30 km depth, 60 km depth, and 150 km depth derived from 3D inversion of AusLAMP MT data (black triangles).

**Fig. 3:**
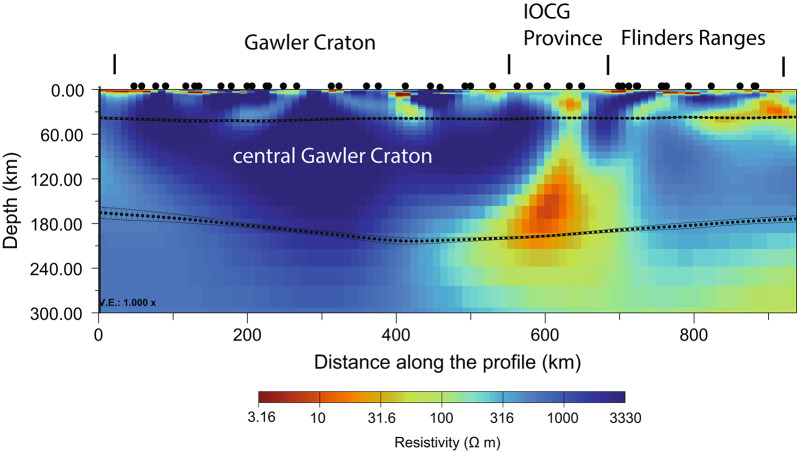
East-west resistivity cross-section derived from 3D inversion of AusLAMP magnetotelluric data to depths of 300 km. Location of the profile is denoted in Fig. 2. Dotted lines indicate the Moho^36^ (top) and lithosphere-asthenosphere boundary^27^ (bottom). The Moho and LAB depths and uncertainties were calculated from the mean and standard deviation of seven evenly spaced parallel profiles within a hundred kilometre corridor around the profile in Fig. 2.

## The resistivity structure derived from AusLAMP MT data

At mid-crustal (20 km) to upper mantle (100 km) depths, the 3D resistivity model shows a predominantly resistive (>10000$\Omega$m) region occupying the central Gawler Craton (Fig. 2) beneath large parts of the c. 1.59 Ga of the Gawler Large Igneous Province (LIP)^37,38^ and the c. 1.62 Ga St. Peter Suite (Fig. 2). The central resistor is surrounded by an extensive (> 1000 km) arcuate-shaped low resistivity zone, about 50 km wide extending from depths of 10 km to 80 km. The conductor extends from the eastern margin of the Gawler Craton, north along the Coober Pedy Ridge, and extending further west following the Karari Shear Zone. In the eastern part of the survey area, the crustal conductivity structure of the Flinders Ranges and Curnamona Province is more heterogeneous^39^ and follows a general linear NNE trend, unlike the arcuate signature along the Gawler Craton margins.

There exists a remarkable correlation between the location of the lower crustal conductors across the Gawler Craton and Curnamona Province and the location of Cu mineral deposits (Fig. 2). The location of the deposits also follows gradients in the Moho depths^15,40,41^, which suggests a causal relationship between rheological weaknesses exemplified by vertical displacement of the Moho providing a locus for magmatic and fluid flux through the lithospheric column^17^.

The co-located sub-vertical crustal conductors extend down to a flat-lying low resistivity zone starting at depths of around 90 km beneath the eastern Gawler Craton and Flinders Ranges and deepening towards the west with its top at around 120 km beneath the central Gawler Craton (Fig. 3). This WNW-ESE trending Gawler Craton mantle conductor is spatially extensive beneath the northern Gawler Craton and extending further east beneath the Curnamona Province (Fig. 2). It was first identified as an anomalous geophysical mantle signature from MT^42^ and has a potential anisotropic signature^43^ of conductive orientation aligned with the mapped mantle conductor derived from NNE-trending phase tensors at periods >1000 s (Supplementary material, Fig. 6), particularly across the more resistive central Gawler Craton due to the larger depth sensitivity. While not the focus of this study, further analyses are warranted to investigate the potential anisotropic nature of the entire Gawler craton lithosphere based on the fits of the modelled phase tensor for long periods in the central Gawler craton in addition to previous modelling^43^. It has been correlated to a few percent reductions in seismic shear wave velocities at a postulated Gawler Craton mid-lithospheric discontinuity, suggesting a metasomatic footprint^8^. The depth of the conductivity anomaly and seismic wave-speed reduction is comparable to other observed MLD’s at around $\sim$80 km to 120 km depth, e.g., in North America from MT studies^44^ and seismic studies^45^. While there are competing interpretations^46^ for the MLD, one of the likely candidates in Australia are amphiboles as a metasomatic product^47^.

The LAB beneath the study area is $\sim$100 km deeper than the top of the Gawler Craton mantle conductor, and extends from 180 km beneath the Curnamona Province to over 220 km in the central Gawler Craton. The prolonged tectonic quiescence since the Proterozoic and the current heat flow of $\sim$54mW$\hbox {m}^{-2}$ to $\sim$75mW$\hbox {m}^{-2}$ in the Gawler Craton and Curnamona Province make partial melts an unlikely explanation for the mantle conductor, with implications for the longevity of the low resistivity zones over time.

The likely cause of the Gawler Craton mantle conductor is, therefore, a mechanism involving the enrichment of nominally anhydrous mantle minerals during a metasomatizing event^48^. The spatial correlation with the Mesoproterozoic Cu occurrences in the study region (Fig. 2) suggests that the resistivity signals reflect geological events dating back to the Proterozoic. The diffusion rates of hydrogen, even accounting for faster grain boundary diffusion, are only on the order of a few tens of kilometers for the temperature conditions prevalent in the Gawler craton mantle^49^. Such slow diffusion rates can explain the survival of the conductivity signature observed at present.

**Fig. 4:**
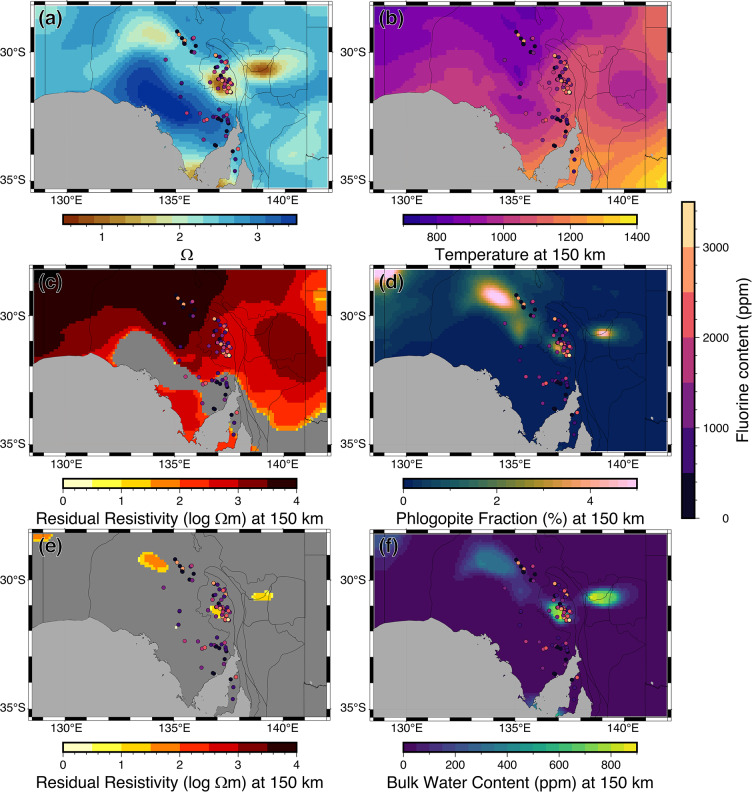
Fitting water and phlogopite content in the mantle at 150 km depth. (**a**) Resistivity value from inversion; (**b**) Present-day temperature derived from a paleogeotherm-constrained seismic tomography model^59^; (**c**) Residual resistivity after fitting a dry lherzolitic composition. The residual is calculated by subtracting the resistivity value from the MT model from the forward resistivity of a dry lherzolite. The result is then presented on a logarithmic scale. High residual resistivity indicates that additional conductive phases are required to explain the low resistivity observed in the Gawler Craton; (**d**) Volume fraction of phlogopite required to reproduce the resistivity value; (**e**) Residual resistivity after fitting a lherzolitic composition with its maximum water content. Locations showing high residual resistivity indicate that additional conductive phases (beyond water) are required to explain the MT results; (**f**) Estimated bulk water content required to fit the resistivity value, constrained by water solubility. An elevated fluorine content at the surface is shown as a colored dot.

However, the low resistivity of the mantle conductor of $\sim$10$\Omega$m at the temperatures and pressures at 100 km cannot be solely explained by the addition of hydrogen in the crystal lattice^18,23^ (Fig. 4). Subduction-related enrichment^8,35^ of the lower SCLM can also involve the addition of CO$_2$-rich fluids released from the subducting slab, which can precipitate as graphite along grain boundaries^50^. This mechanism could explain enhanced mantle conductivity to depths of about 150 km, before graphite transitions into the more resistive diamond phase. However, given the reported instability of thin graphite films at temperatures above 1000 K^51^, it is questionable whether graphite can explain the elevated conductivity of the Gawler mantle conductor.

Another permissible candidate for the reduced resistivity is a minor phlogopite phase due to the enhanced abundance of fluorine associated with the 1.59 Ga Gawler-LIP^35,52^. At the surface above the mantle conductor, Fluorine enabled the extensive flows associated with the Gawler Range Volcanics^53^, one of the most voluminous felsic magmatic events preserved on Earth^38^. The comparable mantle depth of the top of the conductor and the observed shear wave speed reduction in the AusREM data^27^ within the Gawler Craton^8^ suggest a common cause. Seismically slow and electrically conductive phases, such as pyroxenes, amphiboles, and phlogopite, are viable explanations at globally observed mid-lithosphere discontinuity (MLD) between 80 km and 120km^19,46,47^ and act as sinks for first-row transition elements^54^, such as Fe, Cu, Co, and Ni, which are found in the IOCG deposits near the surface^55^. The spatial coincidence and recent measurements of anomalously high electrical conductivity of F$^-$ charge carriers in phlogopite^56^ at mantle conditions make a phlogopite phase a viable candidate^54,57,58^ (Fig. 4). Notably, the presence of halogens (F, Cl) has a unique impact on mantle conditions and can remobilize metals from the deep mantle to the crust, as imaged in our example (Fig. 3).

## The iron-oxide Cu-Au mineral system footprint

In light of the diverse orientations of the crustal and mantle conductors across the survey area, and the tectonic history of the Gawler Craton, the enrichment of Cu and Fe-ore in the IOCG deposits likely involves a two-stage geodynamic process^8,35^. At first, likely subduction-related metasomatism prior or syn-1620 Ma led to the enrichment of the bottom of the continental SCLM beneath the Gawler Craton, which may have occurred at several times over the evolution of the craton, including in the Neoarchean^30^. We note the comparable depths ($\sim$120 km) of the WNW-ESE linear Gawler Craton mantle conductor to modern day backarc partial melt pockets in active ocean-continent collision zones which form above a subducting oceanic slab, e.g. in Northern Cascadia^60^. The geometry of the conductor is permissible for a NEN- or SWS-verging subduction zone with a linear backarc parallel to the current orientation of the conductor. Analogous to the modern subduction zones, we prefer a NEN-verging subduction, which places the isotopically more juvenile St. Peters Suite in the location of the arc/forearc^35,61^, with the highest mantle resistivities due to a depleted lithosphere beneath the subducting slab. From the mantle conductor, Cu- and Fe-rich melts would likely have percolated up into the lower crust in areas of weaker rheology either at the cratonic margin or substantial pre-existing weaknesses within the craton. The footprints of these potential Cu, Fe and sulphide reservoirs^62^ are still visible in the continuous lower crustal conductors imaged in our 3D model, in particular as sulphides are known to significantly reduce electrical bulk conductivity in rocks^63^.

The second significant geodynamic event leading to the IOCG mineral system occurred at c. 1.59 Ga, causing the most significant re-mobilization and re-activation in the history of the Gawler Craton. Magmatic re-organization at that time are seen in the wide-spread emplacement of the Hiltaba Suite granites, host to the IOCG deposits, and the contemporaneous Gawler Range Volcanics. We interpret this event to be largely responsible for tapping the lower crustal Cu reservoirs and transporting mineral-rich magmas to the surface, followed by subsequent hydrothermal alteration leading to the mineral enrichment of IOCG deposits^64^. This event explains the spatial correlation between the IOCG deposits at the surface and the lower crustal conductors.

Our models illustrate the influence of isopycnic cratonic blocks^65^ on guiding the ascent of metal-bearing melts and fluids from subduction-modified SCLM in the Proterozoic, providing first-order control on the location of metallogenic provinces. Such pathways are preserved for more than a billion years as regions of anomalously low resistivity in the sub-continental lithospheric mantle. Regions of low resistivity in the lower crust show remarkable correlation with spatial occurrences of copper in the upper crust suggesting that fluid processes mobilising copper from the mantle and lower crust leave a resistivity signature, potentially due to phlogopite-metasomatized lithosphere and enhanced water content in the mantle and sulphides at grain boundaries in the crust. These new models image the deeper parts of lithospheric mineral systems for the first time, and represent a new approach to mapping metal distributions in the upper crust through the use of the continent-wide AusLAMP-type deployments.

## Methods

### Build resistivity model

The Australian Lithospheric Architecture Magnetotelluric Project (AusLAMP) is the Australian initiative to map the Australian continental lithosphere using magnetotelluric (MT) stations to obtain a resistivity model of the continent. It is a joint project between Geoscience Australia, state geological surveys, and Universities to collect an array of long-period MT sites spaced every half degree latitude/longitude (roughly 50 km).

The 3D inverse model described here is based on 282 long-period ($T=$8 s to 16000 s) MT sites, including high-quality legacy data^42,66^, spatially extending longitudinally from the western to eastern geographical border of South Australia and between 28.5$^{\circ }$S and 35$^{\circ }$S latitude. Instruments recorded naturally occurring electric and magnetic field variations for over three weeks at each site. The time-series data is processed using robust processing codes^67^ to obtain frequency dependent ratios of the electric (MT impedance) and vertical magnetic field (tipper) variations to the horizontal magnetic field variations, respectively. The obtained periodicities of the signal stretches from $\sim$8 s to 16000 s, which corresponds to depths of a few kilometers to several hundred kilometers, in particular within generally resistive Archean lithosphere.

We invert the impedance and tipper data using the 3D ModEM code to obtain the three-dimensional resistivity distribution across South Australia^68,69^. We use error floors of $3\%$ and $10\%$ for the off-diagonal and diagonal impedance tensor components, respectively, and 0.03 for the tipper. The final normalised rms of the 282 stations is 1.92 (supplementary information, Fig. 7). The discretized model domain encompasses an area of 2700 km$\times$3150 km$\times$2360 km discretized into 101$\times$146$\times$82 cells in the $\{x,y,z\}$ direction. The model presented here is the best fitting model out of a suite of over 20 different inversions where we tested various modelling parameters; including covariance matrices, starting half-spaces, error floors and smoothing parameters following approaches used from other AusLAMP work in South Australia^70,71^. The preferred model in Fig. 2 began from a 100$\Omega$m half-space, fixed bathymetry, and ocean sediments added beneath. The conductivity connection beneath the eastern Gawler craton in the vicinity of the IOCG deposits is tested using an embedded resistor in place of the conductive N-S trending crustal and upper mantle anomaly. The results indicate that the phases of tens of stations are affected if the resistor is embedded, suggesting that the model is sensitive to the conductive structure (supplementary information, Figs. 8, 9).

### Estimate lithospheric mantle water and phlogopite content

We used the PIDE^72^ (Petrophysical Interpretation tools for geoDynamic Exploration) to quantify the lithospheric mantle water content and composition based on the conductivity model. PIDE is an expanded version of MATE^73^(Mantle Analysis Tools for Electromagnetics) software, which integrates laboratory electrical conductivity measurements of mantle minerals under high-pressure and high-temperature conditions. Using these experimental parameters, PIDE can forward-calculate electrical resistivity based on mantle composition, temperature, water content, and the interconnection of mineral phases. It can also perform inversion to estimate composition and water content based on temperature and electrical resistivity.

To quantify the nature of the conductive mantle beneath the IOCG province, we use the 3D temperature model of Australia^59^ to account for the thermal effects on the resistivity model, which is derived from a shear-wave velocity model constrained by paleo-geotherm data from mantle xenoliths^47^. We selected representative experimental datasets to calculate the electrical conductivities of olivine^74^, orthopyroxene^75^, clinopyroxene^76^, garnet^77^, and phlogopite^78^. The bulk conductivity was calculated using a Hashin-Shtrikman Upper Bound mixing model^79^. We also performed experiments using different phase mixing models, including the Hashin-Shtrikman lower bound mixing model^79^ and a modified Archie’s Law^80^ with varying interconnectivity parameters (m=1 represents perfect connectivity, while m=2 indicates partial connectivity).

For the initial testing, we fit water content to a dry lherzolite composition. Although there is no direct constraint on mantle composition in the IOCG province, garnet analyses of mantle xenocrysts indicate that lithospheric mantle compositions across kimberlite localities in the southern Gawler Craton are dominantly lherzolitic^47^. The dry lherzolitic also been used to represent mantle compositon for the MT study in the southern Gawler Craton^81,82^. The maximum water content is constrained by the water solubility of each mantle mineral phase. Our analysis shows that the mantle is one orders of magnitude more conductive than a lherzolite mantle with its maximum water content.

**Fig. 5:**
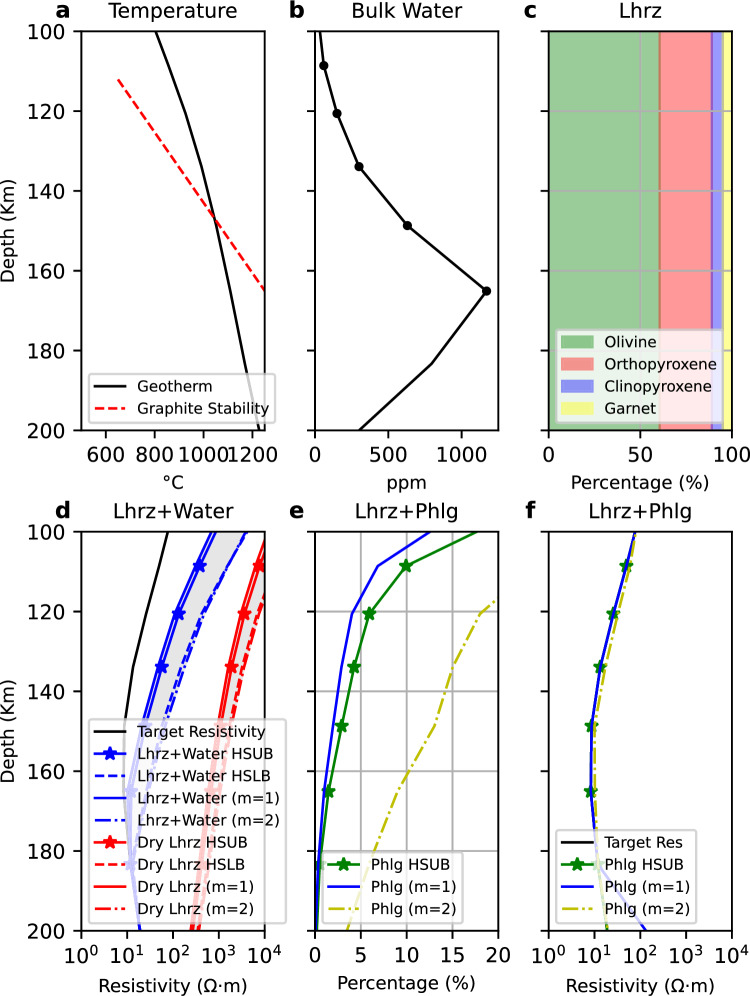
Fitting water and phlogopite (Phlg) content in mantle. (**a**) Temperature profile from a paleogeotherm-constrained seismic tomography model^59^. Dashed line shows the stability field of graphite; (**b**) Maximum water content for a lherzolitic composition constrained by the water solubility; (**c**) Percentage of each mineral phase in a lherzolitic composition; (**d**) Fitting the resistivity profile with a lherzolitic (Lhrz) composition and water, applying different phase mixing laws. The Hashin-Shtrikman upper bound (HSUB) mixing model and the modified Archie’s law with perfect connectivity (m=1) resolve a more conductive mantle. In contrast, the Hashin-Shtrikman lower bound (HSLB) mixing model and the modified Archie’s law with partial connectivity (m=2) resolve a more resistive mantle. In any case, an additional conductive phase is required to fit the MT model; (**e**) Required phlogopite content variation for fitting low resistivity in the mantle; (**f**) Fitting the resistivity profile with a lherzolitic composition and phlogopite.

Given that water alone is insufficient to account for the observed resistivities in the middle lithosphere of the IOCG province, additional conductive phases are required. Considering the extensive fluorine enrichment in this region, we test whether phlogopite can account for the high conductivity (Fig. 5).

We solve the phlogopite content in the mantle assuming its association with a 0.5 wt% fluorine. Although the fluorine content is low, experimental data indicate that it can dramatically enhance mantle conductivity^78^. In particular, conductivity increases linearly with fluorine content^78^. By fitting phlogopite to the mantle, our results show that up to $10\%$ of phlogopite could account for the high conductivity, suggesting that the mantle in this region is likely phlogopite-metasomatized. The presence of wehrlite in mantle xenocrysts from the southern Gawler Craton could further support this interpretation^47^.

## Supplementary Information

Supplementary Information.

## Data Availability

Details about the AusLAMP MT data and corresponding models are available through the Geological Survey of South Australia data portal - https://catalog.sarig.sa.gov.au/dataset/mesac150, including the Gawler craton survey portion presented here (https://catalog.sarig.sa.gov.au/dataset/mesac27800).
