# Supplementary material for: Mantle-derived fluid flux controls Olympic Dam-style Fe oxide-Cu-Au mineralisation
Source: Sci Rep. 2026 Jan 16;16:3444. doi: 10.1038/s41598-025-33477-7 (PMC12835537; doi:10.1038/s41598-025-33477-7)
Supplement: Supplementary file 1 — Supplementary Information. [file 41598_2025_33477_MOESM1_ESM.pdf]

Supplementary information

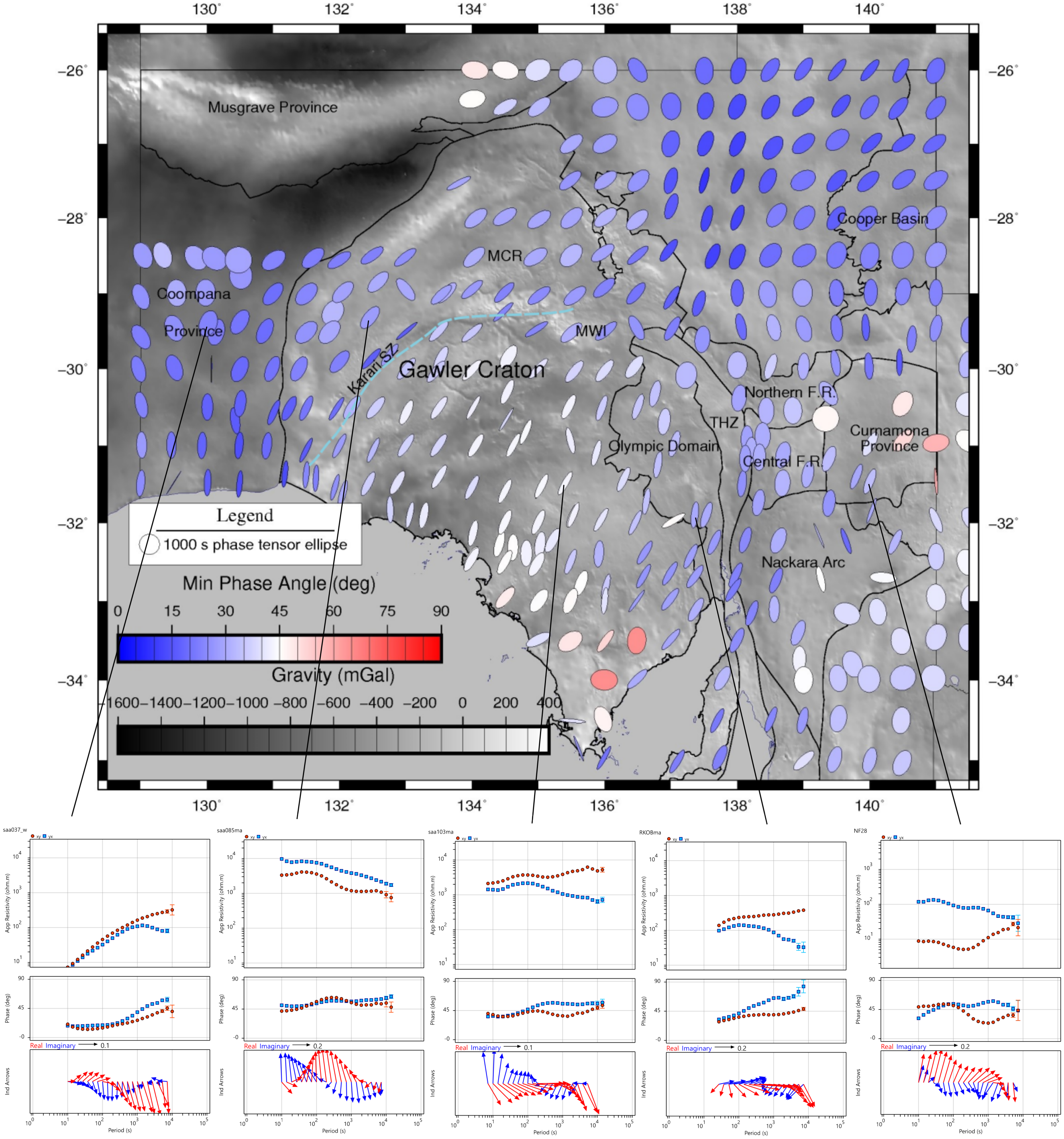

Figure 6. Top: Phase tensors for 1000 s period plotted on gray-scale gravity map of South Australia. The data includes the array presented in this manuscript and MT AusLAMP data in the north-east of South Australia across the Cooper Basin[70]. THZ - Torrens Hinge Zone, MWI - Mount Woods inlier, CPR - Coober Pedy Ridge. Bottom: Key magnetotelluric transfer functions with panels showing apparent resistivity, phases and induction arrows against period from top to bottom. These represent different domains from west to east, with notably higher apparent resistivity in the central Gawler Craton.

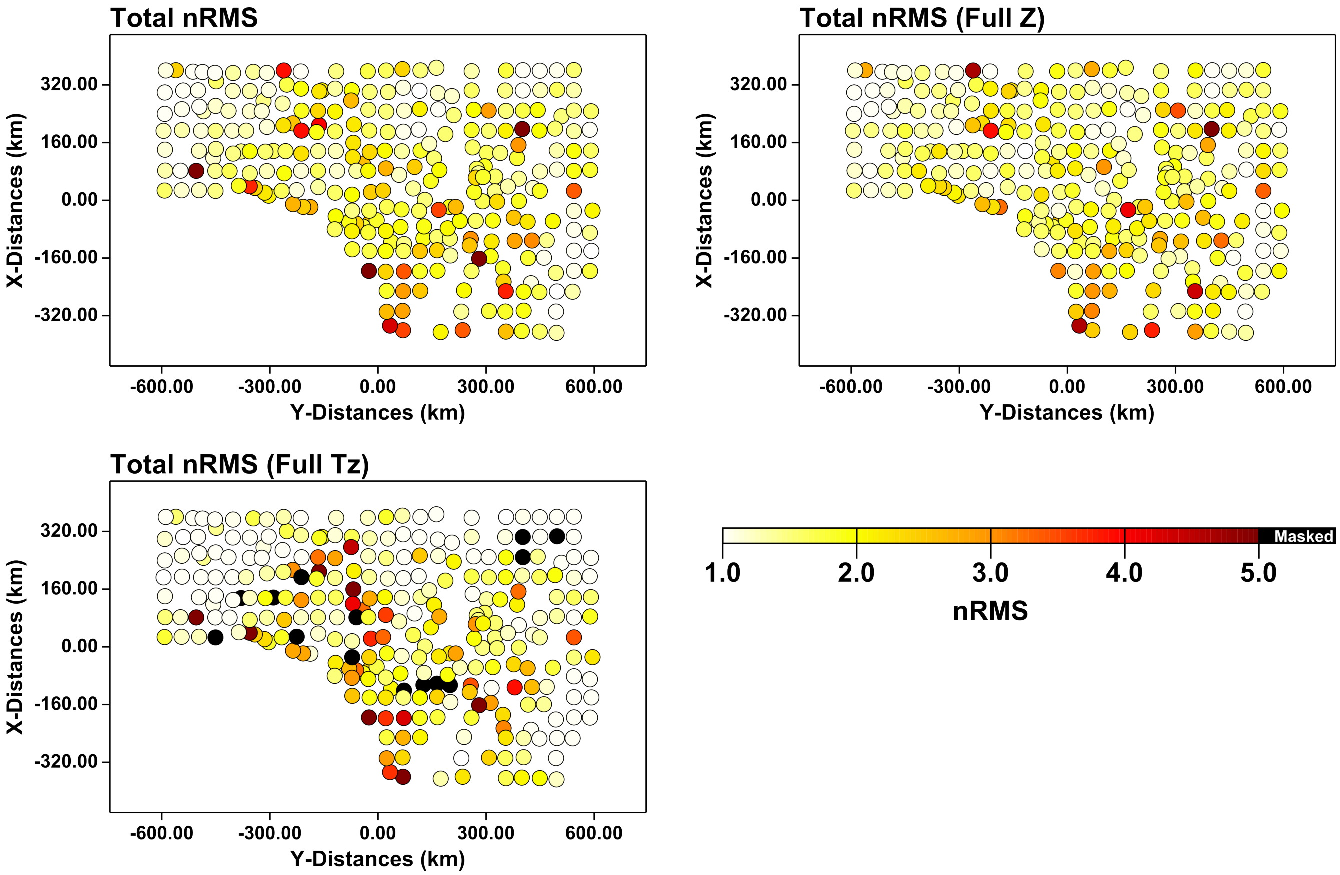

Figure 7. Normalised root mean square (nrms) fits of modelled vs observed MT data constraining the model shown in Figure 2 and 3. Final total nrms is 1.92.

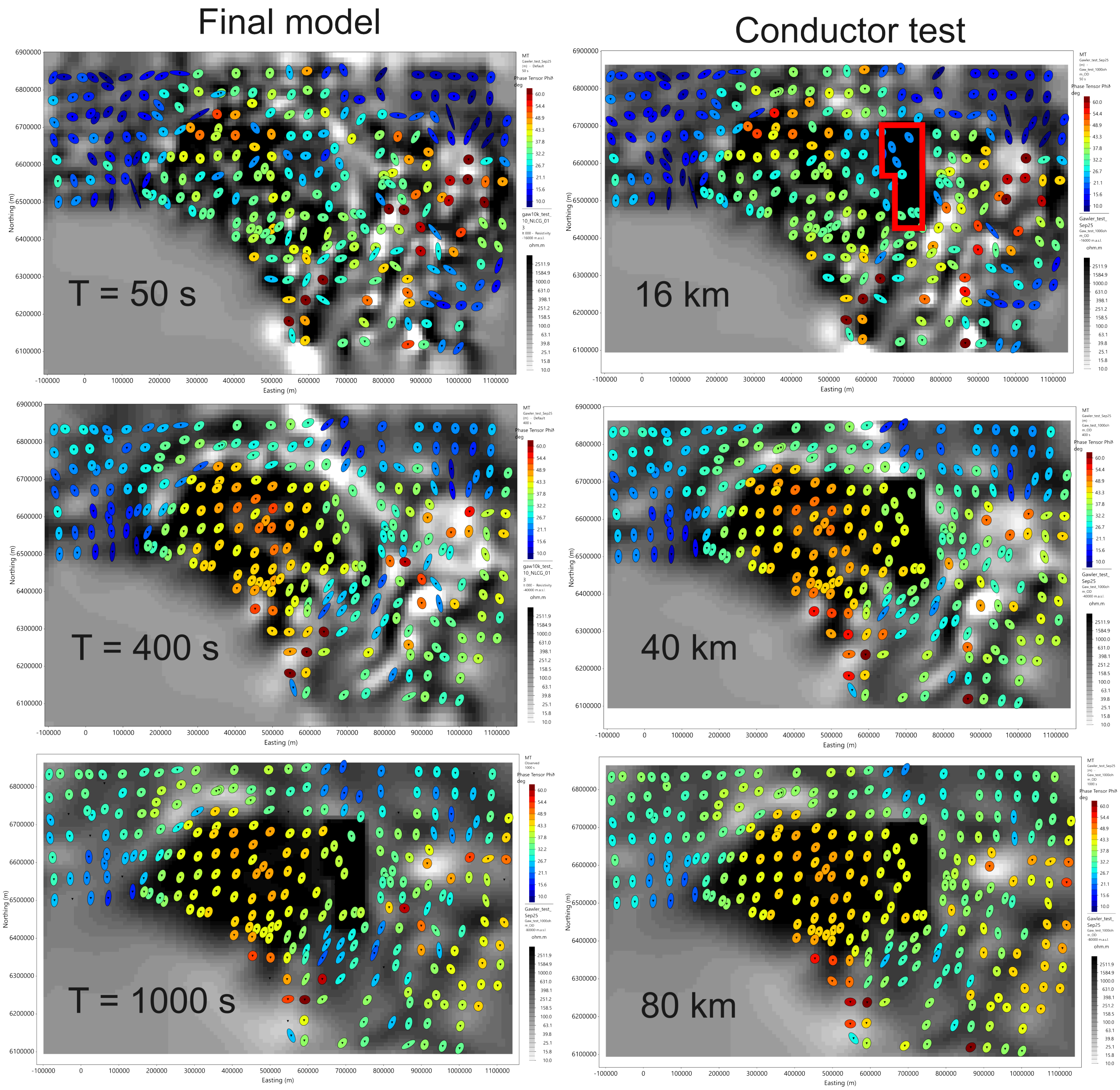

Figure 8. Sensitivity analysis of the crustal and upper mantle conductivity connection beneath the IOCG province of the eastern Gawler craton. A vertical resistor (5000 Ω m) was embedded into the location of the conductivity pathway from the mantle into the crust between depths of 16 km and the top of the mantle conductor at ∼110 km as outlined in red polygon top left. Left panel: final 3D resistivity model and predicted phase tensor responses at three different periods between 50 s and 700 s, overlain on resistivity depth slices at three different depths between 16 km and 80 km. Right panel: Modelled phase tensor responses at the same periods and plotted on the same resistivity depth slices derived from the final resistivity model with the resistor embedded. The results show that the orientation and phase values of the phase tensors change across the eastern Gawler craton, not just above the embedded resistor. Particularly stations atop conductors that connect to the eastern Gawler craton conductivity anomaly in the crust and upper mantle are affected. We conclude that enhanced N-S trending crustal and upper mantle conductivity is required by the model.

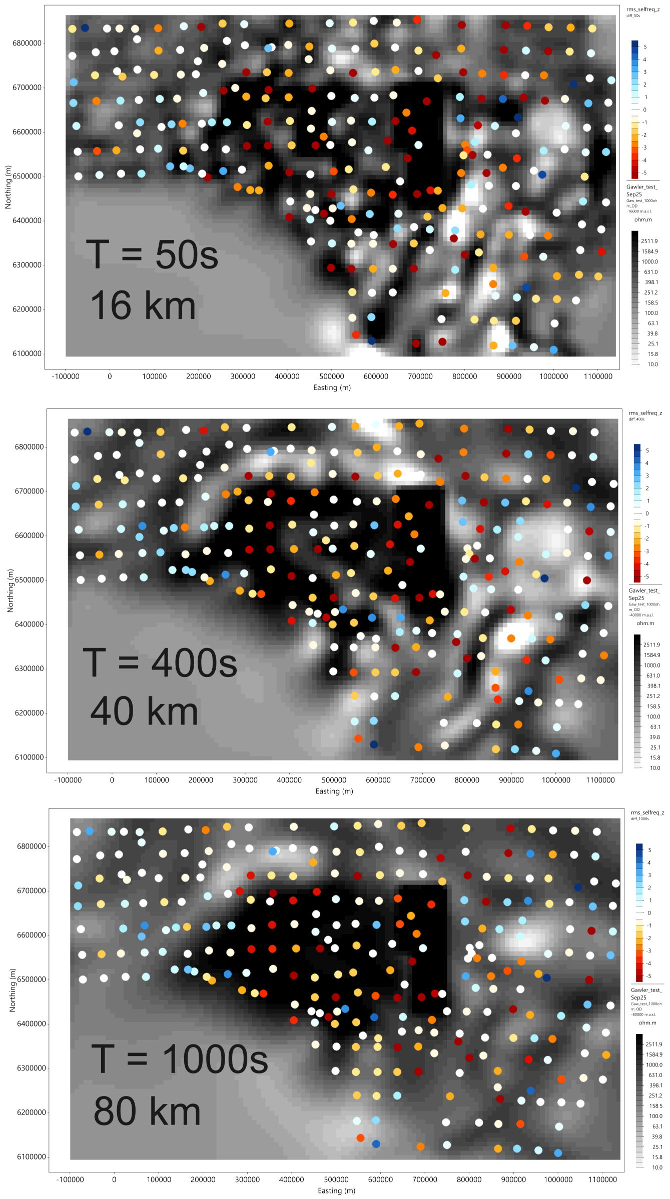

Figure 9. Plots of root mean square (rms) difference corresponding to Figure 7. The tests were performed using the Viridien Geotools software package based on the finite-difference forward modelling and a non-linear conjugate gradient inversion algorithm[82]. The difference is calculated as diff $= Z_{\mathrm{fwd}} - Z_{\mathrm{resistor}}$, albeit with smaller error bars than used in the original inversion using the ModEM inversion[69], resulting in nominally larger rms values. The overall rms across the array increased by 6%. Resulting rms differences are displayed for the same periods as in Figure 7. Negative values or warm colours represent an increase in rms due to the inclusion of a resistor along the eastern margin of the Gawler craton and occur predominantly in the vicinity of the resistor and across the central Gawler craton.
